# Beyond AUC: Clinical interpretability of depression–MRI–machine learning analyses in Alzheimer's disease

**DOI:** 10.1002/alz.71435

**Published:** 2026-04-24

**Authors:** Wenzhi Deng, Yongzhi Xie

**Affiliations:** ^1^ Department of Pathology The Third Xiangya Hospital Central South University Changsha China; ^2^ Department of Radiology The Third Xiangya Hospital Central South University Changsha China

1

Dear Editor,

We read with interest the recent article by Tang et al.^1^ using National Alzheimer's Coordinating Center (NACC) data to characterize depressive symptoms in Alzheimer's disease (AD), relate symptom burden to magnetic resonance imaging (MRI)‐derived volumes, and evaluate machine learning (ML) models for AD classification and depression severity prediction. In 2722 participants (886 AD; 1836 cognitively normal controls), 21.4% met the authors’ threshold for clinically significant depressive symptoms (Geriatric Depression Scale [GDS] ≥ 5), with higher prevalence in AD than controls (35.3% vs. 14.7%). ML discrimination for AD versus controls was strong (random forest area under the curve [AUC] 0.871), and depression severity prediction was moderate (random forest *R* ^2^ 0.625).^1^ The work is timely and clinically relevant, and it provides a valuable foundation for improving neuropsychiatric phenotyping and prediction in AD.

To further support interpretability and downstream translation, future studies building on this framework may consider several design and reporting elements that can help clinicians understand what is being predicted, for whom, and how predictions might change decisions in practice.

First, depression phenotyping in dementia can benefit from triangulation across complementary measures. While a GDS cut‐point is practical, depressive symptoms in dementia may overlap with apathy, sleep/appetite changes, and broader neuropsychiatric symptoms. Future analyses could therefore stratify performance and brain symptom associations by dementia severity (e.g., Clinical Dementia Rating [CDR] strata) and cross‐validate GDS‐defined symptoms against dementia‐tailored measures available in NACC, such as Neuropsychiatric Inventory depression/dysphoria items or clinician diagnosis where available. Evidence syntheses suggest the Cornell Scale for Depression in Dementia (CSDD) has comparatively strong support for detecting depression in dementia settings, and it may serve as a useful external benchmark when feasible.^2^

Second, spectrum effects are central to clinical deployment. The current case–control definition maximizes separation (controls with Mini‐Mental State Examination [MMSE] ≥ 27 and CDR = 0; AD with MMSE ≤ 24 and CDR ≥ 1), but memory‐clinic populations often include boundary states (mild cognitive impairment [MCI] and very mild AD) in which diagnostic uncertainty is greatest. Future work could evaluate whether key brain–depression associations and prediction performance generalize across clinically relevant contrasts (cognitively normal vs. MCI; MCI vs. early AD), and whether within‐AD associations persist after severity stratification. Such analyses can clarify how depressive symptom burden behaves across the disease continuum and how model utility shifts with case mix.^3^

Third, clarifying the causal estimand can strengthen interpretation of adjusted brain–symptom associations. Including MMSE as a covariate may be reasonable for some questions, but cognition can also lie on the pathway between neurodegeneration and mood symptoms, and adjustment may change the meaning of the estimated effect (e.g., approximating a “direct effect” rather than a “total effect”) or introduce collider structures in some settings. Future studies could explicitly state the intended estimand (total vs. direct), provide a brief causal diagram motivating covariate choice, and report sensitivity analyses with and without cognitive adjustment to demonstrate robustness of conclusions.^4^

Fourth, for ML models intended to inform clinical thinking, complementary evaluation beyond discrimination is essential. AUC alone does not indicate whether predicted probabilities are accurate (calibration), how decisions should be thresholded (clinical cut‐points), or whether using the model improves outcomes or resource allocation (net benefit). Future work could align reporting with TRIPOD+AI (Transparent Reporting of a multivariable prediction model for Individual Prognosis Or Diagnosis + Artificial Intelligence) guidance and assess risk of bias and applicability using PROBAST+AI (The Prediction model Risk Of Bias ASsessment Tool + Artificial Intelligence).^5^, ^6^ In addition, decision‐analytic evaluation (e.g., decision curve analysis) can help quantify clinical value across plausible threshold probabilities and show whether the model would meaningfully change decisions compared to default strategies.^7^

Last, outcome distributional features may guide more practice‐facing modeling. The low median GDS scores in both groups (median 1.0 with differing interquartile ranges) suggest skewness and possible zero inflation. Future studies could consider ordinal/count models or two‐part approaches and prioritize clinically interpretable outputs—such as the probability of clinically significant symptoms—alongside continuous severity prediction. This may better align statistical modeling with clinical communication and intervention planning.

In summary, Tang et al. provide an ambitious and informative analysis linking depressive symptoms, neuroimaging markers, and ML prediction in AD. We hope the above considerations—framed as opportunities for future work on depression phenotyping, spectrum generalizability, estimand transparency, and clinically oriented model evaluation—will help strengthen interpretability and accelerate translation into practice and prospective study designs.

## AUTHOR CONTRIBUTIONS

**Wenzhi Deng, Yongzhi Xie**: conceived the ideas and framework for the letter, reviewed the article thoroughly, and drafted the initial manuscript. **Wenzhi Deng, Yongzhi Xie**: contributed to the critical analysis of the article, provided insights into clinical applicability, and assisted in revising the draft for clarity and impact. All authors read and approved the final manuscript for submission.

## CONFLICT OF INTEREST STATEMENT

We declare there are no conflicts of interest. Author disclosures are available in the Supporting Information.

## FUNDING INFORMATION

This work was not supported by any specific funding source.

## Supporting information



Supporting Information

## Data Availability

Not applicable.
